# Perfuorooctane Sulfonate (PFOS), Perfluorooctanoic Acid (PFOA), Brominated Dioxins (PBDDs) and Furans (PBDFs) in Wild and Farmed Organisms at Different Trophic Levels in the Mediterranean Sea

**DOI:** 10.3390/toxics6030050

**Published:** 2018-08-22

**Authors:** Elena Fattore, Renzo Bagnati, Andrea Colombo, Roberto Fanelli, Roberto Miniero, Gianfranco Brambilla, Alessandro Di Domenico, Alessandra Roncarati, Enrico Davoli

**Affiliations:** 1 Department of Environmental Health Sciences, Istituto di Ricerche Farmacologiche Mario Negri IRCCS, 20156 Milano, Italy; elena.fattore@marionegri.it (E.F.); renzo.bagnati@marionegri.it (R.B.); andrea.colombo@marionegri.it (A.C.); roberto.fanelli@marionegri.it (R.F.); 2 Toxicological Chemistry Unit, Environment Department, Italian National Institute of Health, 00161 Rome, Italy; roberto.miniero@iss.it (R.M.); gianfranco.brambilla@iss.it (G.B.); alessandro.didomenico@iss.it (A.D.D.); 3 School of Biosciences and Veterinary Medicine, University of Camerino, I-62024 Matelica, Italy; alessandra.roncarati@unicam.it

**Keywords:** perfluorooctane sulfonate, perfluorooctane acid, PFOS, PFOA, mediteranean fish, toxicological risk

## Abstract

The present study shows the results of perfuorooctane sulfonate (PFOS), perfluorooctanoic acid (PFOA), brominated dioxins (PBDDs) and furans (PBDFs) measured in several marine fish and seafood of commercial interest at different trophic levels of the food chain. The aims were to investigate the level of the contamination in Mediterranean aquatic wildlife, and in farmed fish, to assess human exposure associated to fishery products consumption. Samples of wild fish were collected during three different sampling campaigns in different Food and Agriculture Organization (FAO) 37 areas of the Mediterranean Sea. In addition, farmed fish (gilthead sea bream and European sea bass) from off-shore cages from different marine aquaculture plants. Results showed contamination values of PFOS and PFOA were lower than those detected in sea basins other than the Mediterranean Sea. Concentration values of PFOS were generally higher than those of PFOA; moreover, levels in farmed fish were lower than in wild samples from the Mediterranean Sea. Intake of PFOS and PFOA through fishery products consumption was estimated to be 2.12 and 0.24 ng/kg·BW·day, respectively, for high consumers (95th percentile). Results of 2,3,7,8-substituted congeners of PBDDs and PBDFs were almost all below the limit of detection (LOD), making it difficult to establish the contribution of these compounds to the total contamination of dioxin-like compounds in fish and fishery products.

## 1. Introduction

Perfluorooctane sulfonate (PFOS) and perfluorooctanoic acid (PFOA) are two chemicals included in the large group of the perfluorinated compounds (PFC) which have been widely produced for industrial purposes since 1950 [1]. They are characterized by a fully fluorinated hydrophobic chain and a hydrophilic head and these properties, in combination with their high chemical stability, make these compounds unique for their ability to repel both water and oils. They have been used in many applications, such as surface treatments for coatings, clothes, carpets, packaging products, cookware, food contact papers, and as additives in the fire-fighting foam. They are considered emerging pollutants, since they have been detected in human tissues and wildlife with increasing trends [2,3,4] and seem to meet the criteria of persistence, biomagnification, and long-distance transport, to be included in the persistent organic pollutants (POPs) under the Stockholm convention. Liver is the target organ of toxicity of these chemicals. Toxicity of PFOS and PFOA includes developmental effects, interference with transport and metabolism of fatty acids, immune-suppression, and interference with thyroid hormones. PFOA shows the typical effects of the peroxisome proliferator-activated receptor alpha (PPAR-α) agonists, which include increase of β-oxidation and cytochrome P450 mediated reactions [5]. For both compounds, carcinogenicity has been shown in animal study mediated by a non-genotoxic mechanism. 

PBDDs and PBDFs are another group of POPs formed as byproducts of other brominated compounds, such as brominated flame retardants (BFRs) or brominated biphenyls (PBBs), or ex novo in the combustion processes starting from brominated precursors. In addition, for PBDDs, a biogenic origin in the marine environment has been hypothesized [6]. These compounds are of concern because they seem to have the same mechanism of toxicity of the highly toxic 2,3,7,8-substituted congeners of the polychlorinated dibenzo-p-dioxins (PCDDs) and furans (PCDFs) through the binding to the aryl hydrocarbon receptor (AhR) [7,8], which is the protein mediating the dioxin-like toxicity [9]. Indeed, the classical fingerprint of the dioxin-like biological effects, such as wasting syndrome, thymic atrophy, chloracne, teratogenesis, reproductive effects, and immunotoxicity have been observed [10].

One of the main research gaps related to these pollutants is to what extent exposure for humans and other living organisms occurs [11], since few data on environmental occurrence are available, especially for PBDDs and PBDFs. Fish and fishery products are a known source of dietary intake of POPs for general population, since seas and oceans represent the final accumulation tank of such compounds and their tendency to bioaccumulate.

Within a more extensive research project on the welfare of wildlife and farmed fauna in the Mediterranean Sea [12], this paper reports detailed results species and location specific for PFOS, PFOA, PBBD and PBDF analysis in fish and other aquatic organisms collected in different areas of the Mediterranean Sea. The aims were to investigate the level of the contamination in farmed and wild fish at different trophic levels of the food chain and to assess human exposure associated to fish and fishery products consumption.

## 2. Materials and Methods

### 2.1. Sampling

Wild aquatic organisms, at different levels of the food chain, were collected during different sampling campaigns in May, November and January, in three areas in the Mediterranean Sea. The different sampling areas were selected based on the anthropic level of the coasts and were located close to: Monopoli, in the Adriatic sea, south Bari; Porto Palo, in the Ionian sea, in front of the city of Pachino; and Bagnara Calabra, in the Tyrrhenian Sea nearby the Eolie Islands. During the first two sampling campaigns, farmed fish (gilthead sea bream and European sea bass) from off-shore cages have been also collected from three different aquaculture plants. In total. 61 samples of aquatic organisms were analyzed for PFOS, PFOA, PBDDs and PBDFs. The species analyzed with the corresponding sampling areas are shown in the Table 1.

For fish of larger sizes (>100 g), the fillet was isolated and analyzed, whereas for species of smaller size, where it was difficult to separate the fillet, the analysis was performed on the whole fish without head, tail and entrails. For shrimps, the analyzed samples consisted of the body without exoskeleton.

### 2.2. PFOS and PFOA Analytical Procedure

Fresh samples (0.5 g), after spiking of the internal standard 13C12 PFOS and 13C12 PFOA (Wellington Laboratories, Guelph, ON, Canada) in methanol, were extracted by ultrasounds for 40 min, and centrifugated at 2800 rpm for 10 min. The supernatant (0.5 mL) was transferred to glass vials and added to 0.5 mL Milli-Q water. Instrumental analysis was performed by high pressure liquid chromatography-tandem mass spectrometry (HPLC-MS-MS) Perkin-Elmer Series 200 (Waltham, MA, USA), Applied Biosystem API 3000 (Concord, ON, Canada) with electrospray ionization (ESI). The HPLC conditions were the following: chromatographic column XTerra MS C18 2.1 × 100 mm, 3.5 μm. The mobile phase A was 5 mM ammonium acetate and the mobile phase B was acetonitrile and the flow rate 200 μL/min. Spectrometric conditions have been optimized in multiple reaction monitoring (MRM) mode using a continuous direct infusion of a solution of the analytes. Detailed analytical methodology for PFOS and PFOA quantification will be published elsewhere.

### 2.3. PBDD and PBDF Analytical Procedure

Homogenized samples (20–60 g) were lyophilized (Thermo MicroModulyo Freeze Dryer, Fisher Scientific, Hampton, NH, USA) and spiked with a mixture of the following labeled internal standard: 2,3,7,8-tetrabromodibenzofuran (2,3,7,8-TBDF)-13C12; 1,2,3,7,8-pentabromodibenzo-p-dioxin (1,2,3,7,8-PBDD)-13C12, 1,2,3,7,8-pentabromodibenzofuran (1,2,3,7,8-PBDF)-13C12, 2,3,4,7,8-pentabromodibenzofuran (2,3,4,7,8-PBDF)-13C12, and 1,2,3,4,7,8-hexbromodibenzofuran (1,2,3,4,7,8-HBDF)-13C12. Labeled and native analytical standards (congeners 2,3,7,8-substituted from tetra to hexa for dioxins and furan and 1,2,3,4,6,7,8-heptabromodibenzofuran) were purchased from Cambridge Isotope Laboratories Inc. (Tewksbury, MA, USA). Lyophilized samples were extracted using an accelerated solvent extractor ASE300 (Dionex, Sunnyvale, CA, USA) by a mixture of n-hexane:acetone (9:1) and three extraction cycles using a 60% vessel flush at 80 °C and 1500 psi. The extracts were completely evaporated until dryness by rotary evaporator and the fat content was determined gravimetrically. Cleanup was carried out overnight adding sulfuric acid on an Extrelut column and subsequently by alumina column, adapting the clean-up procedure for chlorinated dioxins.

Quantification has been performed by high resolution gas chromatography–high resolution mass spectrometry HRGC-HRMS (Thermo Fisher, Waltham, MA, USA) using a thermo Finnigan MAT95 XP mass spectrometry with GC PAL, CTC Analytics auto sampler, in EI^+^ and SIM modes, electron energy 38 eV, ion source temperature 280 °C, resolution power 8000–10,000. The selected ions used for quantification and confirmation were M+2 and M+4 for TBDF and TBDD; M+4 and M+6 for PBDF, PBDD, HeBDF and HeBDD; and M+6 and M+8 for HpBDF. The chromatographic conditions were: capillary column J&W DB-5MS, 30 m × 0.25 mm, film thickness 0.1 μm. Temperature program: 80 °C, 25 °C/min until 180 °C; 3 °C/min until 280 °C; 6 °C/min until 310 °C for 7 min. For the limit of detection (LOD), a signal-to-noise ratio of 3:1 was chosen. 

## 3. Results and Discussion

Sampling details and descriptive statistics for complete sample dataset has been reported elsewhere [12]. Concentration values for PFOA were below the LOD (0.05 ng/g fresh weight) in 37 samples and for PFOS in 11 samples, out of 65, corresponding to a total below LOD of 57% for PFOA and 17% for PFOS of negative results, respectively. Levels ranged from <0.05 to 1.89, and from <0.05 to 5.96 ng/g fresh weight (fw) for PFOA and PFOS, respectively. Levels ranged from < 0.05 to 1.89, and from <0.05 to 5.96 ng/g fresh weight (fw) for PFOA and PFOS, respectively. Mean ± standard deviation concentrations for PFOA and PFOS in the wildlife of the three sampling areas of the Mediterranean Sea were, respectively, 0.09 ± 0.11 and 1.19 ± 0.91 in Porto Palo, 0.06 ± 0.08 and 1.27 ± 1.36 in Bagnara Calabra, and 0.20 ± 0.47 and 1.25 ± 1.06 in Monopoli. As already reported in other investigations on levels of these chemicals in aquatic organisms, concentrations of PFOS resulted higher than those of PFOA [13]. In addition, concentration levels measured in this study are markedly lower than those reported in other geographical areas but comparable to those in muscle fish and other aquatic organisms sampled in the Mediterranean Sea [1,14,15]. 

**Figure 1:**
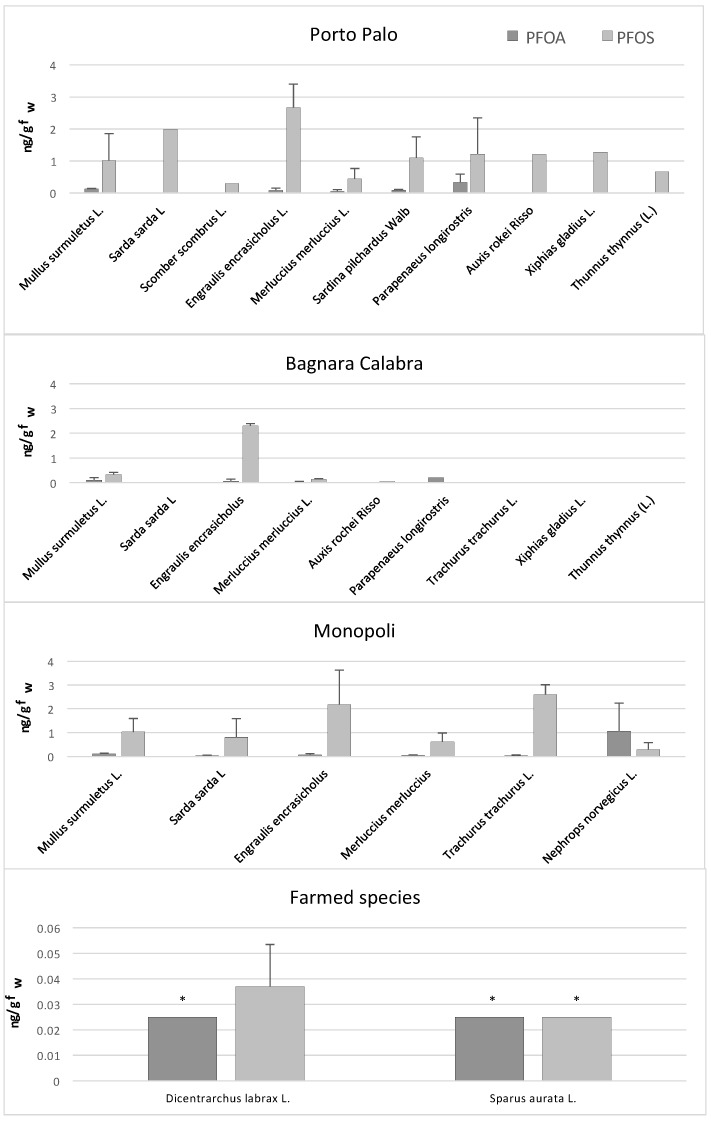
PFOS (perfuorooctane sulfonate) and PFOA (perfluorooctanoic acid) mean concentrations ± standard deviation expressed as ng/g of fresh weight (fw) in wild aquatic organisms from Porto Palo, Bagnara Calabra and Monopoli in the Mediterranean Sea, in farmed fish. * ½ Limit of Detection (LOD).

Figure 1 shows the PFOA and PFOS concentrations in the samples analyzed. From that figure, it is evident that levels of both chemicals in the farmed sea bass (*Dicentrarchus labrax* L.) and sea bream (*Sparus aurata* L.) are at least one order of magnitude lower than those measured in the wildlife of the Mediterranean Sea. These results seem to indicate that contamination from PFOS and PFOA arises from food through biomagnification rather than directly from water (bioconcentration) since the farmed fish analyzed have been sampled in offshore cages of the aquaculture plants. A larger set of samples should be performed in order to provide better statistics, especially if there is an interest in Species-Specific risk assessment. 

**Figure 2:**
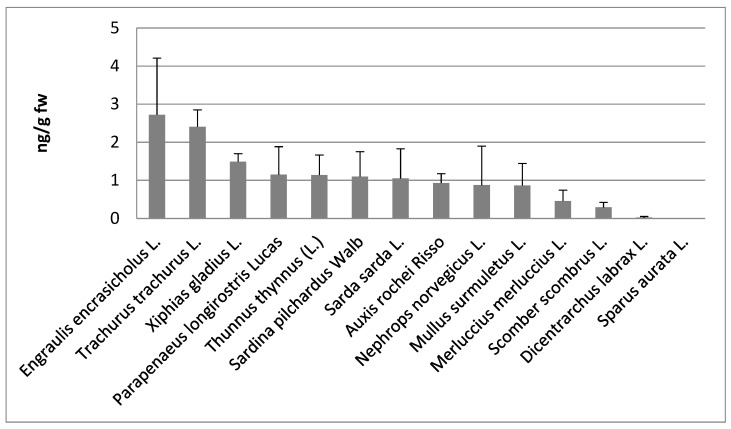
PFOS and PFOA mean concentrations ± standard deviation expressed as ng/g of fresh weight (fw) in different aquatic species from the Mediterranean Sea.

Among the wildlife, the highest concentrations of PFOS were found in anchovy (*Engraulis encrasicholus* L.), 2.7 ± 1.5 ng/g fw, followed from horse mackerel (*Trachurus trachurus* L.), 2.4 ± 0.4 ng/g fw, whereas the lowest in hake (*Merluccius merluccius* L.), 0.46 ± 0.29 ng/g fw, and Atlantic mackerel (*Scomber scombrus* L.), 0.29 ± 0.13 ng/g fw (Figure 2). Thus, from these data, it is not evident that organisms at higher position in the food chain have higher contamination levels of PFOS and PFOA. That could be due to the different behavior of these chemicals in the bioaccumulation process, since PFOS and PFOA do not accumulate into the fat tissues, as the typical POPs, but they bind serum and liver proteins. However, further studies with a greater number of samples should be performed to confirm this hypothesis.

Analysis of the edible part allows estimating the human exposure to these pollutants through fish and other sea food consumption. Combining food consumption data for the Italian general population [16] and average PFOS and PFOA concentration, as measured in the present study, the human intake associated to average fishery products consumption were 0.82 and 0.09 ng/kg BW·day, respectively, whereas the intake associated to high consumers (95th percentile) were 2.1 and 0.24 ng/kg·BW·day, respectively. These latest figures represent 0.01% and 1.3% of the corresponding tolerable daily intake (TDI) for PFOS and PFOA, respectively, established by the European Food Safety Authority (EFSA) in 2008 [17]. However, in 2016, US-EPA issued a RfD of 20 ng/kg·BW·day valid both for PFOS and PFOA [18], while EFSA is re-evaluating its former 2008 opinion on the basis of epidemiological evidences in PFOS and PFOA exposed groups [19]. Under such scenarios, the seafood consumption could cover approximately the 10% of the PFOS tolerable intakes [20].

Results of PBDD and PBDF analysis showed concentration values above the LOD for at least a congener in only in 5 samples out of 65. The congeners detected above the LOD values were the 2,3,7,8-TBDF, 2,3,7,8-TBDD, 1,2,3,7,8-PBDF, 2,3,4,7,8-PBDF, 1,2,3,7,8-PBDD, and 1,2,3,4,6,7,8-HpBDF. Concentration values of these congeners ranged from 0.01 to 0.89 pg/g fw and the highest values were found in a wild species, (*Sarda sarda* L.), collected in the sampling area of Monopoli. 

Overall, these results indicate that contamination level due to the 2,3,7,8-sostituted congeners of PBDDs and PBDFs, if occurs, is in the range of low ppt; moreover, because of the limited number of samples above the LOD, and the relatively high LOD values (range 0.003–4.5 pg/g fw), it is not possible to make a reliable estimation of the contribution of these compounds to the total contamination of dioxin-like compounds in fish and other aquatic organisms.

## Figures and Tables

**Table 1:** Aquatic species, with the corresponding sampling areas analyzed in the present study.

No of Samples	Species	Sampling Area
5	Gilthead sea bream (*Sparus aurata* L.)	Farmed fish
5	European sea bass (*Dicentrarchus labrax* L.)	Farmed fish
9	Red Mullet (*Mullus surmuletus* L.)	PP, BC, MO
9	Anchovy (*Engraulis encrasicholus* L.)	PP, BC, MO
3	Pilchard (*Sardina pilchardus* Walb.)	PP
3	Pink shrimp (*Parapenaeus longirostris* Lucas)	PP
4	Bonito (*Sarda sarda* L.)	PP, BC, MO
2	Mackerel (*Scomber scombrus* L.)	PP
9	Hake (*Merluccius merluccius* L.)	PP, BC, MO
3	Horse mackerel (*Trachurus trachurus* L.)	MO, BC
2	Norway lobster (*Nephrops norvegicus* L.)	MO
3	Bullet tuna (*Auxis rochei* Risso)	BC, PP
2	Swordfish (*Xiphias gladius* L.)	PP, BC
2	Bluefin Tuna (*Thunnus thynnus* L.)	PP, BC

Legend: PP, Porto Palo; BC, Bagnara Calabra; MO, Monopoli.
